# An Unusual Presentation of Bony Outgrowths on the Chest: A Case from Pakistan

**DOI:** 10.7759/cureus.7309

**Published:** 2020-03-18

**Authors:** Khushboo Nusrat, Samar Mahmood, Muhammad Khan, Aleena Khan

**Affiliations:** 1 Internal Medicine, Dow University of Health Sciences, Karachi, PAK; 2 Internal Medicine, Dow University of Health Sciences, Karachi , PAK

**Keywords:** bony outgrowths, recurrent infections, hereditary multiple exostoses, pediatrics, pakistan, case report

## Abstract

We present a case of hereditary multiple exostoses in a male child, who presented to us with bony outgrowths on the chest and recurrent respiratory infections. On questioning, it was revealed that the child had a family history of bony outgrowths, though those affected in his family were not symptomatic. Other causes of recurrent respiratory infections were systematically ruled out, which led us to our conclusion. The treatment of this condition can be either conservative or surgical, but owing to the seriousness of our patient's condition, the preferred option was surgery in this case.

## Introduction

Hereditary multiple exostoses (HME) is a genetic condition that is characterized by the presence of multiple exostoses (also known as osteochondromas), the commonest benign bone tumors [1]. This condition is known to be inherited in an autosomal dominant pattern and has variable penetrance with two genes involved in its manifestation: EXT1 and EXT2. The rarity of this condition is manifested by the fact that its prevalence is approximately one out of 50,000 in the general population [2].

Normally, HME can present with up to 20 exostoses in a patient. A computed tomography (CT) scan done of our patient revealed bony outgrowths on approximately six to seven locations, with nearly all of them being related to the ribs [2]. The bones that are most commonly involved include, in order of frequency; the femur, tibia, fibula and humerus [3]. On the other hand, the ribs are a rare location for the development of osteochondromas in relation with HME, with the incidence being postulated to be as low as 1% [4]. Our patient presenting primarily with rib exostoses makes this case unique from most others of HME.

## Case presentation

A two-year-old boy presented to the outpatient department at a tertiary care hospital in Karachi, with a complaint of multiple swellings on his chest since birth. He was also suffering from a fever and cough since three days and complained of symptoms that indicated respiratory distress for a day. According to the patient’s mother, the swellings were noticed by her when he was born. However, there was no cause for concern until he had turned one year old. In the last three days, the patient developed a fever and cough, and due to worsening condition, he was brought to the outpatient department. On further questioning, we established that the patient’s respiratory complaints were of a recurrent nature. The patient had already presented and been treated for respiratory infection and distress twice the same year, though further evaluation of his chest swellings had not been carried out. Furthermore, the family was told that the child could have a congenital heart disease, but no relevant work-up had been done. The patient had no contact history with tuberculosis (TB), which is a major contributor to such symptoms in our country. The patient did not have any significant surgical, drug, allergy or blood transfusion history. Interestingly, the patient had a positive family history when it came to the bony outgrowths, with the father, a paternal uncle and his paternal grandmother having similar deformities at different sites over their bodies. However, none of them had the outgrowths over their ribs, nor did they have any features of recurrent respiratory infection. The patient’s appetite, sleep and bowel habits seemed to be normal as well. The review of systems was unremarkable.

**Figure 1:**
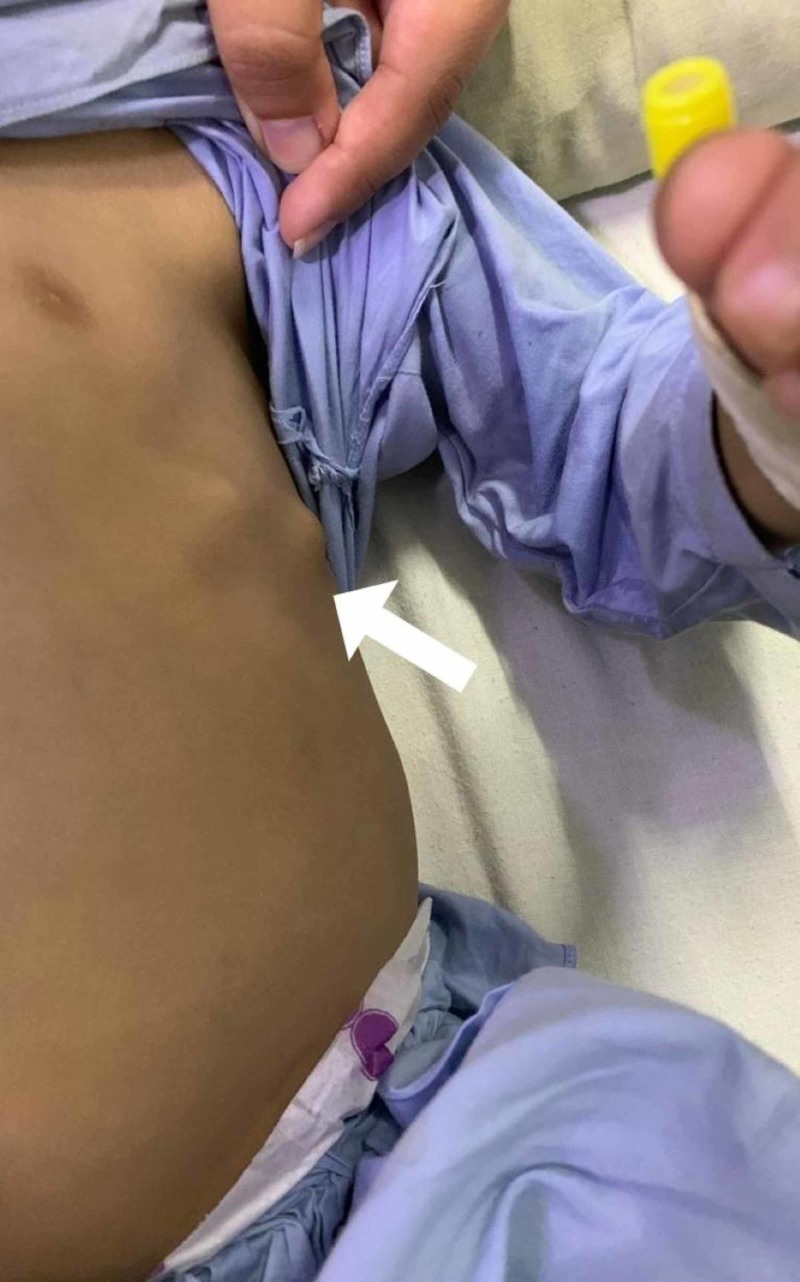
Bony outgrowths on the patient's chest

**Figure 2:**
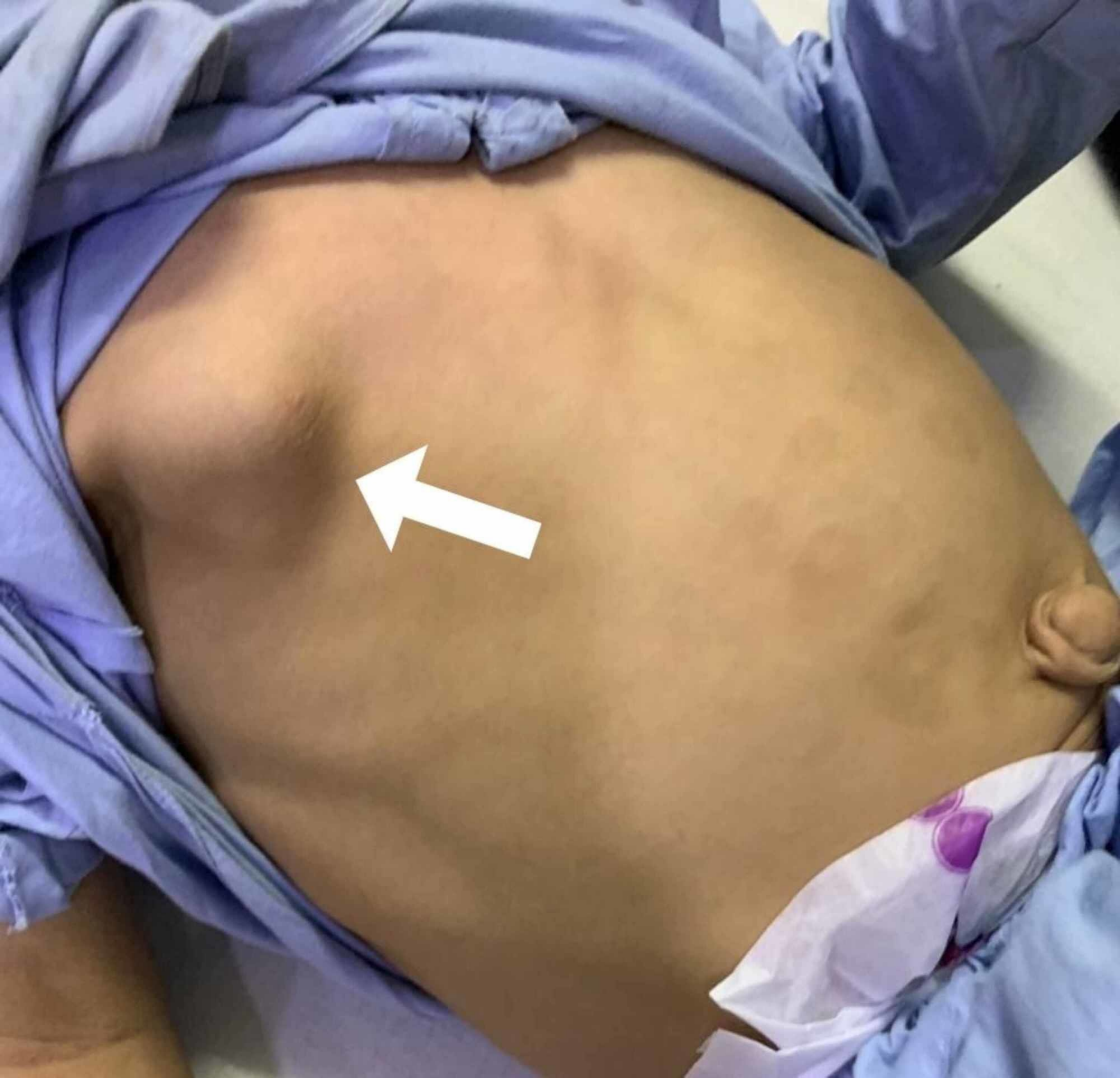
Bony outgrowths on the patient's chest

**Figure 3:**
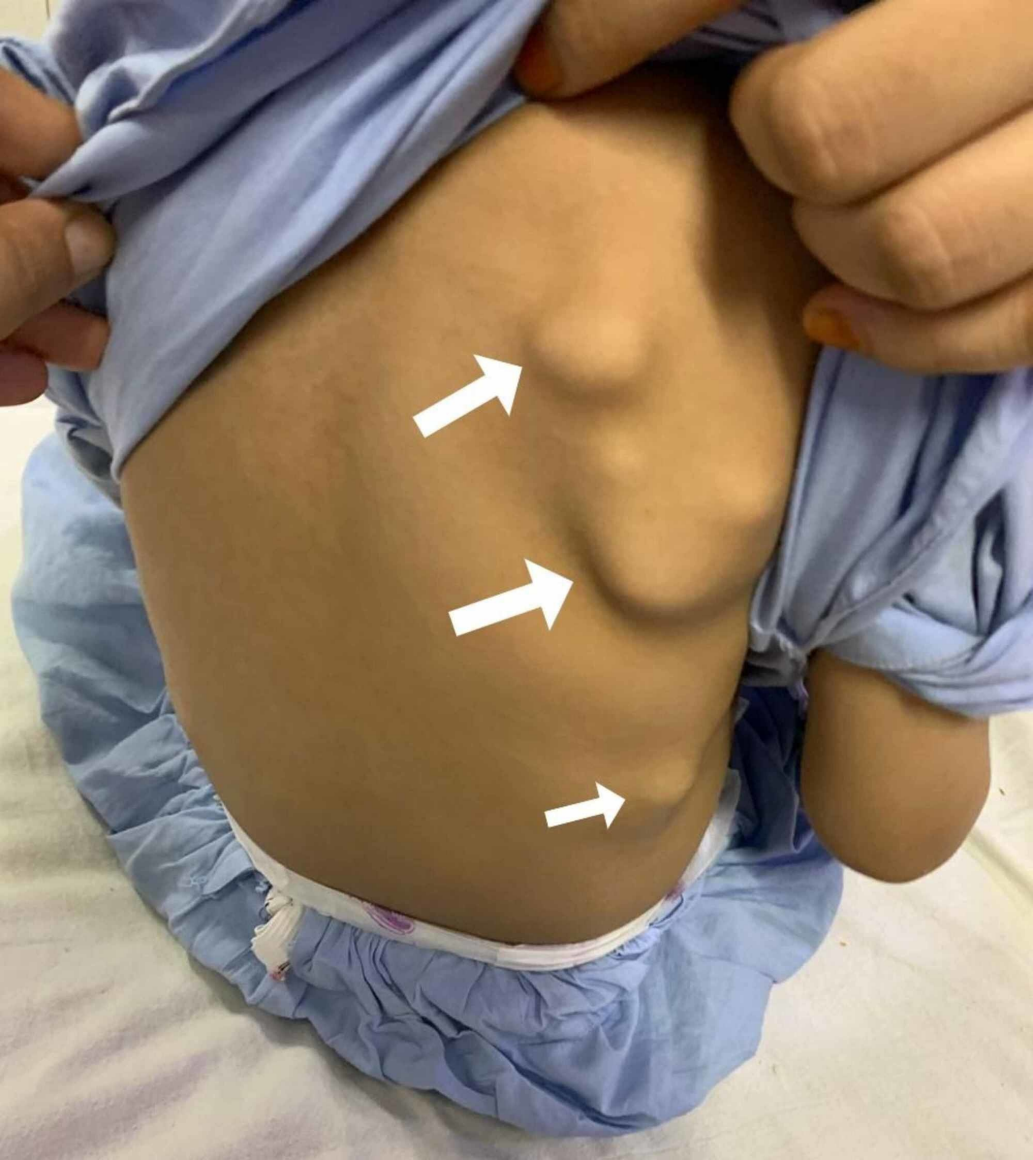
Bony outgrowths on the patient's back

On presentation, the child was irritable, and his respiratory rate was 50 breaths/min. His temperature was also raised, measuring 102°F. Other vital signs were normal. On examining the chest, multiple non-tender swellings were noticed bilaterally, which seemed to be originating over the ribs, anteriorly and posteriorly (Figures 1-3). There was dullness to percussion in the lower zones of the lung, and crepitations were also noticed on auscultation in the same region. Owing to the recurrent nature of his respiratory condition, and the yet to be evaluated chest swellings, the patient was admitted for further evaluation.

 

**Figure 4:**
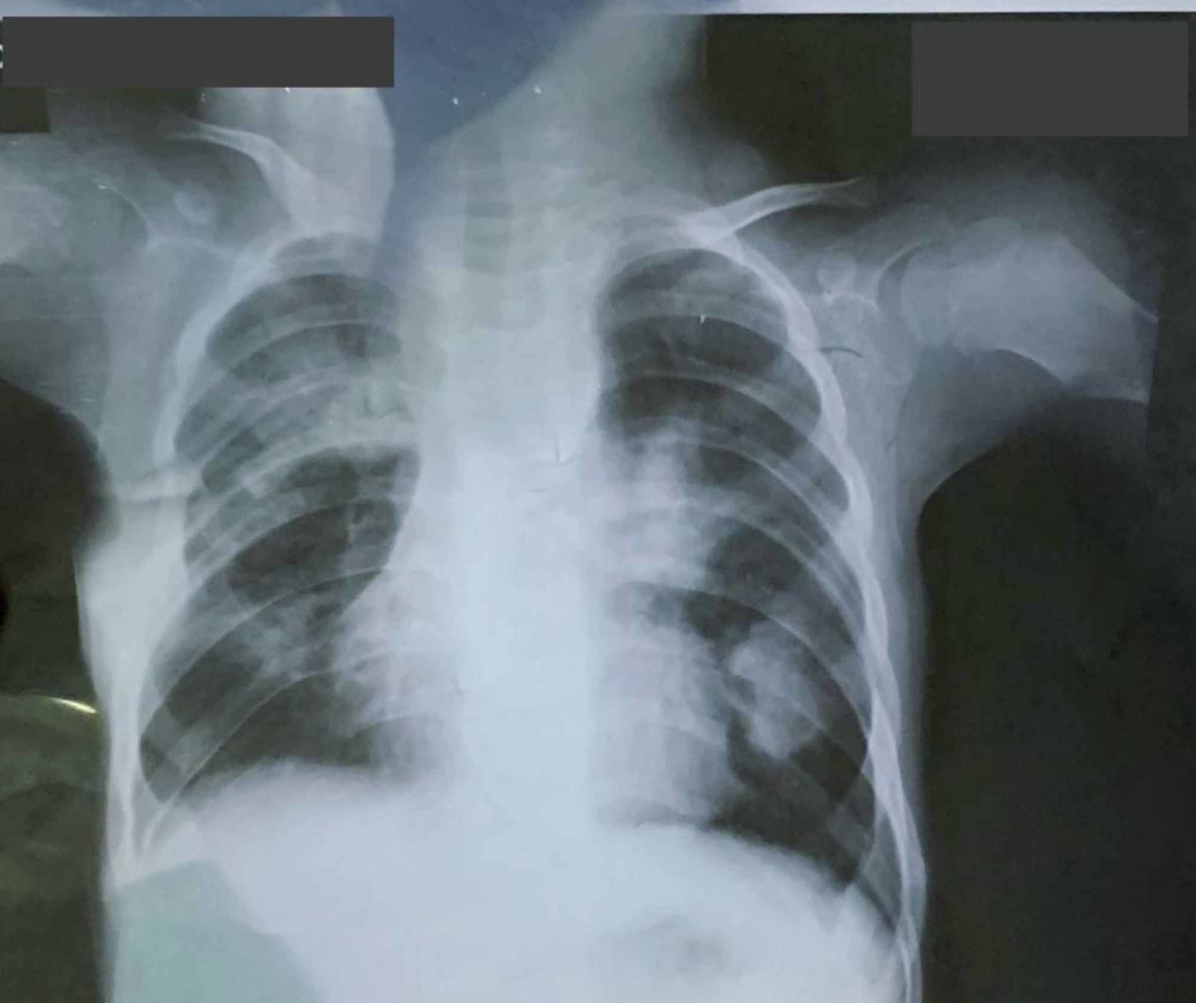
Chest X-ray of the patient before surgery

The patient’s sputum and blood were sent to be cultured to identify the causative organism, which was leading to pneumonia. A chest X-ray was also carried out to aid us in reaching a definitive diagnosis, which revealed bilateral consolidation patches, left-sided lung fibrosis, left lateral chest wall indrawing, lymphadenopathy and mediastinal widening (Figure 4). We carried out the relevant tests to rule out the differentials. For TB, the sputum was sent for culture and staining, and a GeneXpert was carried out as well. Primary immunodeficiency syndromes were ruled out by measuring serum immunoglobulin levels. The recurrent pneumonia also urged us to work up the patient for cystic fibrosis. However, both the sweat test and the genetic studies came out negative. Type 1 diabetes was also ruled out based on random and fasting blood sugar levels. To assess the patient for congenital heart disease, we carried out an echocardiogram. This did not show a congenital abnormality but demonstrated an ejection fraction of 40%, along with a dilated left ventricle, which could be due to the recurrent infections. To assess the bony outgrowths on the chest, we ordered a CT scan which revealed exostoses on the left third, fifth and sixth ribs, and right second, third, fourth and sixth ribs as well as on the medial border of the scapulae, bilaterally. The CT scan supported the chest X-ray findings which revealed multiple homogenous opacities, along with hyperinflated lung fields. Based on such evidence, we were able to diagnose this case as being one of HME, leading to recurrent respiratory infections.

A diagnosis of HME leading to recurrent respiratory infections was made based on the previously mentioned investigations. After admission, the patient was administered a penicillin antibiotic given eight-hourly. The patient was nebulized every six hours as well, as managing his respiratory distress due to the respiratory infection was our primary goal. The patient was examined regularly to note his progress and by the fifth day, our patient was free of fever, cough and chest congestion. The relevant investigations were carried out, and once the diagnosis was reached, our patient was referred to the orthopedic department where his resection would be managed. The operation was carried out within a month of his admission and three months later, he showed no signs of developing a respiratory infection again. Informed written consent was obtained from the patient and his family, granting us permission to report on his condition and the relevant images.

## Discussion

According to a review on the condition, most cases of HME are asymptomatic. However, variable clinical manifestations are seen in this condition, with pain being a relatively frequent occurrence due to compression of tendons and muscles, chronic irritation of surrounding structures or bursitis. Other presentations include limb shortening, coxa valga, brachydactyly and genu valgum, all depending on the location of the exostoses, which could also be variable [5]. Though exostoses of the ribs are asymptomatic in most cases as well, the close proximity to the lung, heart and diaphragm can lead to several complications. More commonly, pneumothorax and hemothorax are encountered, both of which can prove to be life-threatening [6,7].

Another complication, seen less commonly than the two mentioned earlier, is recurrent pneumonia due to costal exostoses [8]. Our patient, along with presenting with exostoses seen solely in the ribs, presented with this rare complication. The presence of the exostoses was noted soon after his birth and he had been treated for pneumonia twice already with the current illness being the third within the year. A similar case in the past revealed, on histological examination of the patient, an empyema and adhesions in relation with an exostoses, a phenomenon that could very well be the cause of the recurrent pneumonia in our patient [9]. Interestingly, our patient's father, uncle and grandmother had similar bony outgrowths but never had any cause for complaint, something which is common in this rare condition. Fortunately, the parents mentioned this during the history-taking, a point that aided us significantly in reaching the diagnosis. Often, parents do not reveal a positive family history, despite having the condition, owing to the latter being asymptomatic [10]. This can hinder diagnoses, as HME is an autosomal dominant condition, with one of the parents always suffering from the condition [2].

Normally, a diagnosis can be made based on clinical and radiological investigations, though chest X-rays are not enough to identify the lesion. However, a CT scan will almost always make the diagnosis, with histological evaluation and genetic testing being supplementary forms of investigation, used only at times of uncertainty [2]. In our patient, the clinical history, including the positive family history, and the radiological investigations were enough to make the diagnosis.

Treatment of the condition depends on several factors. To begin with, possibly the most feared long-term complication of HME is malignant transformation to a chondrosarcoma, a condition that can be difficult to treat [2]. Furthermore, spontaneous hemorrhage due to hemothorax made certain researchers recommend a prophylactic resection, even in asymptomatic cases [11]. The occurrence of long-term complications such as these makes it evident that prophylactic resection is an option which several surgeons recommend and practice [12]. However, resection is not always carried out though periodic radiological evaluations are carried out to rule out the risk of malignancy. These include a CT scan and an MRI, with the latter being far more superior when it comes to identifying malignant transformation. The reason for this is the effectiveness with which an MRI scan can measure cartilage cap thickness, the usual indicator of malignant transformation with a thickness greater than 2 cm being enough to signal the malignant change [13]. For further confirmation, a positron emission tomography scan can also be carried out. In cases such as our patient’s, where the exostoses were leading to recurrent pneumonia, surgery was naturally preferred over conservative management. Several approaches to surgical resection have been developed, with video-assisted thoracoscopy being identified as one of the most commonly used procedures [9].

## Conclusions

HME can lead to recurrent respiratory infections, along with other life-threatening conditions such as hemothorax and pneumothorax. Furthermore, although a familial disease, it is not symptomatic in most cases, as was seen in our patient’s family members who seemed to have similar outgrowths for a significant time, but had no manifestations of the disease. With advances in surgery, HME is a condition that can be potentially cured, effectively eliminating the above-mentioned complications from arising.
